# Human Papillomavirus Persistence in Young Unscreened Women, a Prospective Cohort Study

**DOI:** 10.1371/journal.pone.0027937

**Published:** 2011-11-23

**Authors:** Channa E. Schmeink, Willem J. G. Melchers, Albertus G. Siebers, Wim G. V. Quint, Leon F. A. G. Massuger, Ruud L. M. Bekkers

**Affiliations:** 1 Department of Obstetrics and Gynaecology, Radboud University Nijmegen Medical Centre, Nijmegen, The Netherlands; 2 Department of Medical Microbiology, Radboud University Nijmegen Medical Centre, Nijmegen, The Netherlands; 3 Department of Pathology, Radboud University Nijmegen Medical Centre, Nijmegen, The Netherlands; 4 DDL Diagnostic Laboratory, Voorburg, The Netherlands; Hannover Medical School, Germany

## Abstract

**Objective:**

To evaluate hr-HPV persistence and associated risk factors in a prospective cohort of young unscreened women. Additionally, the relation between hr-HPV status and cytology/histology results is examined.

**Methods and Principal Findings:**

Two year follow-up of 235 out of 2065 young women (18–29 years), participating in a large, one year epidemiological study, with questionnaires, self-collected cervico-vaginal samples (Vibabrush), and SPF_10_LiPA for HPV detection. Only women hr-HPV positive at sample month 12 were invited for a second year of follow-up. After study follow-up, available cytology/histology data were requested from PALGA (the national network and registry of histo- and cytopathology in The Netherlands). These data were compared with available cytology/histology data of the month 12 hr-HPV negative women from the same cohort. 44.1% of the hr-HPV types detected at study month 12, persisted during follow-up. HPV types 45, 31, 16 and 18 were most likely to persist with percentages of 60.0%, 56.8%, 54.4%,and 50.0%, respectively. Compared to newly detected infections at month 12, infections present since 6 months or baseline had an increased risk to persist (OR 3.09 [95% CI: 1.74–5.51] and OR 4.99 [95% CI: 2.67–9.32], respectively). Other co-factors influencing persistence were, multiple HPV infections, smoking and multiple lifetime sexual partners. The percentage of women with a HSIL/CIN2+ (12.1%) in the persistent HPV group, was not significantly different (p = 0.107) from the 5.3% of the women who cleared the hr-HPV infection, but was significantly (*p 0.000*) higher than to the 1.6% of women in the hr-HPV negative control group.

**Conclusion:**

We showed that HPV genotype, multiple infections, smoking, and multiple lifetime sexual partners are co-factors that increase the risk of hr-HPV persistency. Most importantly, we showed that hr-HPV infections are more likely to persist the longer they have been present and that women with a persistent hr-HPV infection have a high risk of HSIL/CIN2+ development.

## Introduction

It has been well established that a human papillomavirus (HPV) infection is a necessary cause in the development of cervical intraepithelial neoplasia (CIN) and cervical cancer [1]–[3]. HPV appears to be the most common sexually transmitted infection and about 80% of all sexually active women will acquire an HPV infection during their life time [2]. Fortunately, only a small proportion of these infections lead to CIN and cervical cancer. A persistent high risk (hr) HPV infection is necessary for the development of cervical carcinoma. Therefore the detection of a persistent hr-HPV infection represents an important marker of an increased risk of CIN and cervical carcinoma [4]–[6]. However, there is no consensus on the definition of persistence.

Most investigators define a persistent HPV infection as detection of the same HPV type, or group of types, on two consecutive visits, but these could be from 2 months up to 72 months apart [7], [8]. Several studies have shown that most infections become undetectable within 1–2 years [5], [9]–[11]. Additionally, infections lasting more than 1 year appear to be associated with a lower clearance rate [5], [9]. Therefore it is more informative to monitor the duration of the infection rather than the number of positive tests [12].

Several risk factors have been identified that are associated with HPV persistence. Especially viral characteristics like viral load, and HPV genotype are linked to persistence [13], [14]. Given that HPV16 and 18 are the most carcinogenic HPV genotypes, it would be useful to know whether their risk of persistence differs from other hr-HPV genotypes [15]. Additionally, some authors have shown that multiple HPV infections are associated with an increased duration of high-risk HPV infections [9], [16], whereas others have failed to show such an increase [11], [17]. Other cofactors like age, condom use, smoking, long-term use of oral contraceptives, high parity, number of sex partners, and exposure to other sexually transmitted diseases have also been associated with HPV persistence [9], [18], [19].

In this study, a prospective cohort of young unscreened women (18–29 years) in the pre-vaccine era was followed for 2 years, to examine the influence of viral factors (i.e. duration of infection, HPV-type and co-infection), and co-factors (i.e. sexual behavior and smoking) on hr-HPV persistence. Additionally we examined the relation between hr-HPV status and follow-up cytology/histology results.

## Materials and Methods

A large prospective cohort study on HPV prevalence, incidence and clearance was performed in the Netherlands, in 2007–2010. At study entry, 2065 unscreened women 18 to 29 years of age were included [20]. Exclusion criteria were, being pregnant, or not fluent in Dutch. Of the 2065 women initially included, 1871 (90.6%) completed the first year of follow-up. Women, who were hr-HPV positive at month 12 (n = 257 (13.7%)), were invited to participate in a second year of follow-up in order to study hr-HPV persistence. In total 235 (91.4%) of the invited women, completed the second year of follow-up and were included in this analysis of persistence. The study was closed after the sample at month 24, therefore all the hr-HPV positive women were referred to their general practitioner or gynaecologist for additional follow-up or treatment. Written informed consent was obtained from all participants. This study was approved by the Local Medical Ethics Committee.

### Specimen collection and HPV DNA detection and genotyping

The women provided five self-samples with a 3-month interval (month 0, 3, 6, 9, and 12) during the first year of the study. In the second year of follow-up, women provided two additional self-samples with a 6 month interval (month 18 and 24). All women received the self sample kit and the additional questionnaires by mail.

The self sample kit contained an explanatory letter, a questionnaire, an illustrated instruction form on how to perform the cervico-vaginal self-sample, a small brush in a sterile cover (Rovers Vibabrush®, Rovers Medical Devices, Oss, the Netherlands), and a collection tube containing medium (SurePath^tm^, Tripath Imaging®, Inc., Burlington NC, U.S.A.), as described previously [20], [21].

Broad-spectrum HPV DNA amplification was performed using a short-PCR-fragment assay. Extracted DNA was used for PCR amplification with the SPF_10_primer sets [22], [23]. The samples were run through an HPV DNA enzyme immunoassay (DEIA) to obtain an OD reading, and categorized as HPV DNA negative, positive, or borderline. The same SPF_10_ amplimers were used on SPF_10_-DEIA-positive samples to identify HPV genotype by reverse hybridization on a line probe assay (LiPA) (SPF_10_HPVLiPA_25_, version 1; Labo Bio-Medical Products B.V., Rijswijk, the Netherlands), which detects the following HPV genotypes: low-risk HPV types: type 6, 11, 34, 40, 42, 43, 44, 53, 54, 55, 66, 70, 74, and “X” (DEIA positive and LiPA negative samples); and high-risk HPV types 16, 18, 31, 33, 35, 39, 45, 51, 52, 56, 58, 59, 68, 73, and 82.

### Type-specific hr-HPV; clearance, persistence, and history

An HPV infection was considered cleared when a woman had two consecutive HPV negative samples [11], [24], [25]. Therefore, hr-HPV positive women with one intermittent type-specific hr-HPV negative sample were considered persistent for that hr-HPV type. All women included in the second year of follow-up were hr-HPV positive at sample month 12, thus only women who were negative for hr-HPV at sample month 18 and 24 were considered to have cleared their hr-HPV infection. (see table 1)

**Table 1:** Definitions of duration of type-specific hr-HPV detection.

	Study sample month					
Type-specific hr-HPV history	Month 0	Month 6	Month 12*	Month 18	Month 24	Type-specifc hr-HPV during 2^nd^ study year
Detected since baseline	**+**	**+**	**+**			
	**+**	**−**	**+**			
Detected since study month 6	**−**	**+**	**+**			
Newly detected at study month 12	**−**	**−**	**+**			
				**−**	**−**	Clearance
				**+**	**−**	Persistence
				**+**	**+**	
				**−**	**+**	

*only women positive for hr-HPV at sample month 12 are included in the 2^nd^ study year and in the analysis of HPV persistency.

The sampling interval in the 2^nd^ study year was 6 months and persistence is generally defined as two consecutive HPV positive samples. Thus, a types–specific hr-HPV infection was considered persistent when it was detected for at least 6 months. Women who were positive at sample month 18 and/or month 24 were therefore considered to have a persistent type-specific hr-HPV infection in the 2^nd^ study year (see table 1). Four of the 235 women were treated for abnormal cytology (and one cervical carcinoma) and cleared hr-HPV before sample month 18, therefore the natural course of clearance or persistence was disturbed. The data of these women were excluded in the analysis of persistence.

To analyze whether the duration of the type-specific hr-HPV infection (the history) prior to sample month 12 influences persistence in the 2^nd^ study year, we determined at which sample moment the hr-HPV infection was first detected. In order to calculate with the same time interval between the samples in the 1^st^ and the 2^nd^ study year, the cut-off point for first detection of the type-specific hr-HPV were made at baseline (month 0), sample month 6 and sample month 12 (see table 1). This analysis was performed for the total number of type-specific hr-HPV infections.

### Hr-HPV status related to cytology and histology

The study protocol was based on self-sampling for HPV detection. Gynecologic histo- and cytopathology data were thus not available at the end of the study. To be able to evaluate the relation between HPV status and cervical lesions, we requested information from PALGA (the national network and registry of histo- and cytopathology in The Netherlands) [26]. To compare data of the hr-HPV positive women included in the 2^nd^ study year, a control group was selected from the women who were hr-HPV negative at sample month 12. These women were 27–29 years at study baseline, and had received an invitation of the Dutch national cervical cancer screening program. In order to relate cytology/histology results to the hr-HPV status, information of all sample points in the 1^st^ and 2^nd^ study year had to be available. The four women who were treated for abnormal cytology before sample month 18, were also excluded in the analysis of persistence related to cytology. This resulted in 224 women hr-HPV positive at sample month 12 and 336 women hr-HPV negative at sample month 12 (control group). PALGA data on gynecologic histo- and cytopathology, test date, and age of the woman at time of the test, were available for 166 (74.1%) hr-HPV positive women and 246 (73.2%) women of the control group. Due to privacy legislation, identification data were made anonymous by a Third Trusted Party (ZorgTTP, Houten, The Netherlands). To relate these anonymous gynecologic histo- and cytopathology test results with the hr-HPV status, women were grouped.

1. Month 12 hr-HPV positive women:
  - type-specific hr-HPV type persistent in the 2^nd^ study year
  - Cleared type-specific hr-HPV type in the 2^nd^ study year
2. Control group: Month 12 hr-HPV negative women

### Statistical analysis

The baseline characteristics of the study population were analyzed and presented in frequencies, mean and standard deviation (SD). The percentage and 95% confidence interval (95% CI) of the hr-HPV prevalence at sample month 12 and persistence in the second year of follow-up were calculated using the number of infections instead of the number of women. Data from the questionnaires were used to determine, with simple logistic regression analysis, whether co-factors like OCP use, smoking and sexual behavior influenced the persistence in the 2^nd^ study year. To analyze the relation between hr-HPV status and histo- and cytopathology test results significance was calculated with the Fischer's exact test. All statistical analyses were performed using SPSS version 16.0 (SPSS Inc., Chicago, IL).

## Results

The 235 hr-HPV positive women included in this analysis had a mean follow-up time of 25.3 months (SD 1.5) and were all born in the Netherlands. At study baseline these women were 18-29 years of age with a mean age of 24 years (SD 3.2), and 233 (99.1%) women reported to ever have had sexual intercourse. Sixty six (28.1%) women reported that they were current smokers, and there were 165 (70.2%) current oral contraceptive pill (OCP) users. These baseline data are shown in table 2.

**Table 2:** Baseline Demographics.

		Sample size N (%)
Total		235
Age (years),mean (SD)	18	24 (3.2)
	19	8 (3.4)
	20	16 (6.8)
	21	19 (8.1)
	22	16 (6.8)
	23	25 (10.6)
	24	15 (6.4)
	25	25 (10.6)
	26	24 (10.2)
	27	18 (7.7)
	28	33 (14.0)
	29	23 (9.8)
		13 (5.5)
Ethnicity	Dutch	235 (100)
	Other	0 (0.0)
Education	Lower secondary/Lower vocational training	5 (2.1)
	Higher secondary/Vocational training	46 (19.6)
	Higher vocational training/University	184 (78.3)
Current Smoking	Yes	66 (28.1)
	No	169 (71.9)
Using OCP	Yes	165 (70.2)
	No	70 (29.8)
Ever had sexual intercourse	Yes	233 (99.1)
No		2 (0.9)

In order to be included in the 2^nd^ study year, women had to be hr-HPV positive at sample month 12. Of the 235 women, 163 had one hr-HPV type, 54 had two hr-HPV types, 15 had 3 hr-HPV types, two had 4 hr-HPV types and one woman had 6 hr-HPV types at sample month 12. This resulted in a total of 330 type-specific hr-HPV infections detected at sample month 12. HPV 16 had the highest prevalence of 30.2%, followed by HPV 51 (19.1%), HPV 31 (16.6%), and HPV 52 (14.9%). HPV 18 was prevalent in 11.1% of the women. (see table 3)

**Table 3:** Ranking of type-specific hr-HPV prevalence at month 12 and type-specific hr-HPV persistence in 2^nd^ study year.

Rank	HPV type	Prevalence at month 12		HPV type	persistence in 2^nd^ study year *	
		N	% (95% CI)		N	% (95% CI)
**1**	16	71	30.2 (24.4–36.5)	45	6/10	60.0 (26.2–87.8)
**2**	51	45	19.1 (14.3–24.8)	31	21/37	56.8 (39.5–72.9)
**3**	31	39	16.6 (12.1–22.0)	16	37/68	54.4 (41.9–66.5)
**4**	52	35	14.9 (10.6–20.1)	18	13/26	50.0 (29.9–70.1)
**5**	56	28	11.9 (8.1–16.8)	33	5/10	50.0 (18.7–81.3)
**6**	18	26	11.1 (7.4–15.8)	56	12/26	46.6 (26.6–66.6)
**7**	39	25	10.6 (7.0–15.3)	39	10/25	40.0 (21.1–61.3)
**8**	68	21	8.9 (5.6–13.3)	68	7/19	36.8 (16.3–61.6)
**9**	33	12	5.1 (2.7–8.7)	52	12/34	35.3 (19.7–53.5)
**10**	45	11	4.7 (2.4–8.2)	35	2/7	28.6 (3.7–71.0)
**11**	59	9	3.8 (1.8–7.1)	51	11/44	25.0 (13.2–40.3)
**12**	35	8	3.4 (1.5–6.6)	59	2/9	22.2 (2.8–60.0)
	Total	330	140.4%	Total	139/315	44.1 (38.6–49.8)

*Four of the 235 women were treated for abnormal cytology before sample month 18, therefore the natural fluctuation of clearance and persistence was disturbed. The data of these women were excluded in the analysis of persistence in the 2^nd^ study year.

The percentage of the type-specific hr-HPV infections, present at sample month 12, persisting in the 2^nd^ study year are shown in table 3. For 315 of the 330 type-specific hr-HPV infections data were available on persistence. Of these 315 type-specific infections, 139 (44.1%) infections were persistent in the 2^nd^ study year. HPV 45 was the most persistent type, with a persistence rate of 60.0%, followed by HPV 31 (56.8%), HPV 16 (54.4%), and HPV type 18 (50.0%). Notably, HPV type 45 was only number 10 in prevalence with 11 (4.7%) type-specific infections at month 12, whereas HPV type 51 (second in prevalence with 45 infections) was one of the least persistent infections with a persistence rate of 25.0%.

Not only the hr-HPV type, but also the duration of the infection prior to sample month 12, seemed to influence persistence in the 2^nd^ study year. Overall, a type-specific infection detected since baseline had an odds ratio (OR) of 4.99 (95% CI: 2.67-9.32) to persist in the 2^nd^ study year, compared to infections newly detected at month 12. Infections first detected at sample month 6 also had an increased risk, OR of 3.09 (95% CI: 1.74-5.51), to persist in the 2^nd^ study year, compared to newly detected infections. (see table 4)

**Table 4:** Influence of the duration of type-specific hr-HPV detection on persistence in 2^nd^ study year.

Type-specific hr-HPV history	N	persistent N (%)	OR (95% CI)	P-value
Detected since baseline	63	43 (68.3)	**4.99 (2.67-9.32)**	**0.000**
Detected since study month 6	70	40 (57.1)	**3.09 (1.74-5.51)**	**0.000**
Newly detected at month 12	166	50 (30.1)	**1 (ref)**	

Having multiple HPV infections at sample month 12, irrespective whether these infections were only hr-HPV or also lr-HPV types, increased a woman's risk to have a type-specific hr-HPV persistent infection in the 2^nd^ study year. This was almost a twofold increased risk, OR 1.93 (95% CI: 1.14–3.26). (See table 5)

**Table 5:** Woman's risk of having a persistent type-specific hr-HPV type in the 2^nd^ study year.

		N* (n = 231)	Persistent N (%)	OR (95% CI)	P-value
Age	Mean: 24.0 years	231	122 (52.1)	1.07 (0.99–1.16)	0.106
	SD: 3.16				
Education	Lower secondary/Lowervocational training	5	3 (60.0)	1.31 (0.21–8.04)	0.769
	Higher secondary/Vocational training	45	23 (51.1)	0.92 (0.48–1.76)	0.898
	Higher vocationaltraining/University	180	96 (53.3)	1 (ref)	
Current Smoking	No	166	81 (48.8)	**1 (ref)**	
	Yes	64	41 (64.1)	**1.87 (1.03–3.39)**	**0.039**
OAC at T12	No	75	36 (48.0)	0.72 (0.41–1.25)	0.240
	Yes	151	85 (56.3)	1 (ref)	
Age at first sexual intercourse	≤ 13	8	6 (75.0)	2.57 (0.37–17.83)	0.339
	14–16	119	61 (51.3)	0.90 (0.29–2.84)	0.859
	17–19	90	48 (53.3)	0.98 (0.31–3.15)	0.972
	≥ 20	13	7 (53.8)	1 (ref)	
Sexual Age∧	Mean: 7.5	228	122 (52.6)	1.07 (0.99–1.16)	0.197
	SD: 3.37				
Lifetime sexual partners	1	15	3 (20.0)	**1 (ref)**	
	2–5	87	45 (51.7)	**4.29 (1.13–16.26)**	**0.032**
	6–10	73	46 (63.0)	**6.82 (1.76–26.33)**	**0.005**
	>10	55	28 (50.9)	**4.15 (1.05–16.34)**	**0.042**
New sexual partner during study period?	No	55	35 (63.6)	1.75 (0.90–3.43)	0.103
	Yes	102	51 (50.0)	1 (ref)	
Type of relationship at study month 12	Married or Living together	47	28 (59.6)	1.47 (0.73–2.98)	
	Couple, living apart	83	44 (53.0)	1.13 (0.63–2.03)	0.281
	Single	98	49 (50.0)	1 (ref)	0.686
Number of sexual partners past 3 months atstudy month 12	0	32	14 (43.8)	1.36 (0.33–5.59)	669
	1	161	86 (53.4)	2.01 (0.57–7.12)	0.281
	2	26	18 (69.2)	3.94 (0.89–17.37)	0.070
	≥3	11	4 (36.4)	1 (ref)	
Sexual frequency past 3 months at T12	1–3	42	26 (61.9)	1.49 (0.68–3.26)	0.318
	4–12	25	10 (40.0)	0.61 (0.24–1.55)	0.299
	13–27	61	35 (57.4)	1.23 (0.62–2.47)	0.552
	>27	69	36 (52.2)	1 (ref)	
Condom use at study month 12	Never	101	60 (59.4)	1.13 (0.45–2.81)	0.800
	Sometimes	39	18 (46.2)	0.66 (0.23–1.86)	0.431
	Most of times	34	16 (47.1)	0.68 (0.24–1.98)	0.484
	Always	23	13 (56.5)	1 (ref)	
Having multiple infections at study month 12? #	Yes	107	66 (61.7)	**1.93 (1.14–3.26)**	**0.015**
	No	123	56 (45.5)	**1 (ref)**	
Other STI during study period?	No	209	112 (53.6)	1.27 (0.52–3.12)	0.602
	Yes	21	10 (47.6)	1 (ref)	

SD: standard deviation.

*Number  =  based on available data, as not all 231 women completely filled-in the questionnaire.

∧Sexual age  =  years of sexual activity (current age minus age of first sexual intercourse).

# Multiple infections  =  irrespective whether these infections were only hr-HPV or also lr-HPV.

Furthermore, table 5 shows that smoking and number of lifetime sexual partners are co-factors influencing hr-HPV persistence. Smokers have an almost twofold increased risk of persistence than non-smokers (OR: 1.87 [95% CI: 1.03–3.39]) and women with multiple lifetime sexual partners at baseline have and increased risk of persistence compared to women with 1 lifetime sexual partner. The OR's for 2-5, 6-10 and more than 10 lifetime sexual partners were 4.29 [95% CI: 1.13–16.26, 6.82 [95% CI: 1.76–26.33] and 4.15 [95% CI: 1.05–16.34], respectively. Of the 15 women with 1 lifetime sexual partner at baseline, only three had a type-specific persistent infection during follow-up. Of the three persistent infections in women with 1 life time sexual partner, one (HPV 16) was newly detected at month 12, and two (HPV 16 and HPV 56) were detected since month 6.

Twelve of the 15 women with 1 lifetime sexual partner at baseline reported to have a new sexual partner during the study follow-up. Together they had 16 type-specific hr-HPV infections detected at sample month 12, of whom 10 were newly detected, four were first detected at month 6, and two at baseline. Because most hr-HPV types were newly detected at month 12 and newly detected hr-HPV infections are less likely to persist, this might be a confounding factor. However, confounding could not be ruled out with multiple logistic regression analysis because ‘duration of hr-HPV detection’ and ‘number of lifetime partners’ are based on different dependent variables, ‘number of infections’ and ‘number of women’, respectively.

The following variables; age, OCP use, age of first sexual intercourse, sexual age, having a new sexual partner during study follow-up, current type of relationship, current number of sexual partners, current frequency of sexual intercourse, current condom use and having a STI during study follow-up, did not influence persistence of a type-specific hr-HPV infection in the 2^nd^ study year. (see table 5).

### Hr-HPV persistence related to cytology and histology

The results from PALGA on cytology and histology related to the hr-HPV status of the women are presented in table 6. The mean age of the women with available cytology results was 28,4 (SD: 2.8). It was not possible to measure the exact time interval between sample month 12 and first cytology because the cases were made anonymous. However, all dates of self-sampling and cytology were available. We calculated the time interval between the date when 50% of the women had taken their self-sample en when 50% of the women had their cytology performed. For the study group women (1a and 1b) this interval was 13 months and for the control group women (2a and 2b) the interval was 12 months.

**Table 6:** Histo- and cytopathology follow-up results.

	Cytology		Histology				
	classification	n (%)	Normal	CIN 1	CIN 2	CIN 3+	No histology
**1. Study group** *							
**a.** Persisting type specific hr-HPV (n = 91)							
	No cytology	1 (1.1)	1				
	Normal	55 (60.4)					55
	ASCUS	16 (17.6)	4	2	1		9
	LSIL	10 (11.0)	1	6	1		6
	HSIL	9 (9.9)			3	5	1
**b.** Cleared type-specific hr-HPV (n = 75)							
	Normal	52 (69.3)					52
	ASCUS	15 (20.0)	3	1			11
	LSIL	5 (6.7)	1	1	1	1	1
	HSIL	3 (4.0)		1	1	1	0
**2. Control group** #							
(n = 246)							
	Normal	227 (92.3)					227
	ASCUS	14 (5.7)	1				13
	LSIL	2 (0.8)			1		1
	HSIL	3 (1.2)				2	1

*Study group: study month 12 hr-HPV positive women. Mean interval between study month 12 and cytology = 13 months.

# Control group: study month 12 hr-HPV negative women. Mean interval between study month 12 and cytology = 12 months.

In the study group 1a; 11 (12.1%) of the 91 women with a persistent hr-HPV infection were identified with HSIL/CIN2+. Four of the 75 women (5.3%) in group 1b, who cleared their type-specific hr-HPV infection in the 2^nd^ study year had HSIL/CIN2+. This difference between both groups was, not significant (*p 0.107*). In the control group, 4 of the 246 women (1.6%) developed HSIL/CIN2+. Women with a persistent hr-HPV type were significantly more often diagnosed with HSIL/CIN2+ compared to women in the control group (*p 0.000*).

## Discussion

In this 2-year prospective cohort study among young unscreened women, we showed that an already persistent hr-HPV infection has an increased risk to persist during follow-up compared to newly detected infections. Hr-HPV types persistent for 6 and 12 months or longer had a respective, threefold or fivefold, increased risk of persistence. Other studies also reported that HPV infections are more likely to persist the longer they have been present [4], [5], [11], [14], [27]. Of the newly detected infections 70% were cleared before the next sample (6 month interval). This is a slightly higher clearance rate than reported in some other studies, who found a mean time to clearance of 6-8 months for newly acquired HPV infections [9], [28].

Persistence was also influenced by the genotype. The overall persistence of type-specific hr-HPV in this study is 44.1%, HPV types 45, 31, 16, and 18, had the highest rate of persistence and the highly prevalent HPV type 51 had a low tendency to persist. In agreement with our findings, other studies also showed that HPV 16, 18, 31, and 45 have an increased risk to persist compared to other HPV types [11], [17], [29]–[32]. Additionally, the IARC multicentre case-control study showed that HPV 16, 18, 45 and 31 were the most common HPV types in cervical carcinoma [33].

Having multiple HPV infections is a risk factor for hr-HPV persistence. The risk to have a persistent type-specific hr-HPV infection during follow-up increased almost twofold in women with multiple (high-risk and/or low-risk HPV) HPV types at month 12. These results are in agreement with other studies showing that women with multiple infections have a higher risk to have a persistent type-specific HPV infection during follow-up [9], [16], [18]. A possible explanation may be that the multiple infections increase the overall viral load of the infection which by sheer volume overcomes immune control. Whether this only happens in women with a lower immunity or also in the general population is not well known. Another explanation may be that there are specific interactions between different HPV genotypes. The impact of having multiple infections on persistence, however, probably ceases in time. Cuschieri et al., showed that over a period of two to three years multiple hr-HPV infections did not constitute a higher risk factor for the development of cervical neoplasia compared with single hr-HPV infections [34].

Only two co-factors, smoking and number of lifetime sexual partners, were found to correlate with the likelihood that a hr-HPV type would persist. Smokers had a two-fold increased risk of hr-HPV persistence. Smoking is a known immune suppressor and a risk factor for cervical carcinoma [35]. However, some studies did not find a difference in HPV persistence between current and non-current smokers [27], [36]. In our study only smoking at baseline was registered, therefore we could not identify differences in the risks between current or past smokers, nor for the duration of smoking, or number of cigarettes a day.

Women with 1 lifetime sexual partner at baseline were at lower risk for a persistent infection than women with multiple lifetime sexual partners. The highest risk was found for women with 6–10 lifetime sexual partners at baseline. It should be noted, however, that the majority of the women with 1 lifetime sexual partner at baseline, had a new sexual partner during study follow-up and newly identified hr-HPV types. Newly detected hr-HPV types are less likely to persist, so this might be a confounding factor. In literature, the number of lifetime sexual partners is not a consistent risk factor for hr-HPV persistency.[36], [37] Therefore, the potential influence of the number of lifetime sexual partners on hr-HPV persistency still needs to be elucidated.

The use of OCP's is also a potential risk factor for the development of cervical carcinoma [38]. However, results from our study and others did not find a relation between OCP use and persistence [21], [27], [36]. This increased risk is reported to be strongly related to the duration of use and the effect proved reversible after cessation. This influence of OCP use on HPV persistence, however, is still controversial [35].

Previously it has been shown that women with a persistent type-specific hr-HPV infection are significantly more likely to have or develop CIN than those who were sequentially infected by different hr-HPV types or who cleared their infection [34], [39], [40]. We also found that having a persistent type-specific hr-HPV infection is associated with a higher (not significant) rate of HSIL/CIN2+ development (12.1%) compared to women who cleared the type-specific hr-HPV infection (5.3%), and significantly higher (*p 0.000*) compared to women who were hr-HPV negative at study month 12 (1.6%).

Due to the anonymous histo- and cytopathology data, we were not able to correlate the histo- and cytopathology results to the type-specific hr-HPV types, nor if the woman had multiple HPV types. Therefore we could not confirm whether the infections with HPV types 45, 31, 16, and 18 were more likely to cause HSIL/CIN2+ and whether the number of infections was correlated to the severity of the lesion as described previously [41].

There is considerable interest in the possibility of using HPV testing as a primary cervical cancer screening tool [42], [43]. Our data support that following prevalently detected hr-HPV types for persistence is useful to identify women with an increased risk of CIN in the following years [44]. In order to do so, a clear definition of a clinically relevant persistent HPV infection should be determined. This definition should be based on the duration of the type-specific hr-HPV presence that is predictive of CIN development rather than based on two consecutive hr-HPV positive visits.

Based on our results in this population of young women, we suggest that this interval should be a minimal of 6 months, because a 6 month persistent hr-HPV type had a threefold increased risk to persist during follow-up compared to a newly detected hr-HPV infection. However, this interval may be extended to 12 months, because 68.3% of the hr-HPV types already persistent for 12 months or longer continued to persist during follow-up. Therefore a 12 month interval for detection of type-specific hr-HPV persistency will select a group of women that needs close surveillance for HSIL/CIN2+ development in the following year(s).

In conclusion, we showed that co-factors increasing the risk of hr-HPV persistency are, genotype specific (45, 31,16, and 18, are most likely to persist), multiple infections, smoking, and multiple sexual lifetime partners. Most importantly, we showed that hr-HPV infections are more likely to persist the longer they have been detected and that women with a persistent hr-HPV infection have a higher rate of HSIL/CIN2+ detection in the following year. Thus, women with a persistent hr-HPV infection should be monitored for HSIL/CIN2+ development.
